# Applying generalized additive models to unravel dynamic changes in anthocyanin biosynthesis in methyl jasmonate elicited grapevine (*Vitis vinifera* cv. Gamay) cell cultures

**DOI:** 10.1038/hortres.2017.38

**Published:** 2017-07-26

**Authors:** Nay Min Min Thaw Saw, Claudio Moser, Stefan Martens, Pietro Franceschi

**Affiliations:** 1 Research and Innovation Centre, Fondazione Edmund Mach (FEM), Via E. Mach 1, San Michele all'Adige 38010, Italy

## Abstract

Plant cell cultures represent important model systems to understand metabolism and its modulation by regulatory factors. Even in controlled conditions, cell metabolism is highly dynamic and can be fully characterized only by time course experiments. Here, we show that statistical analysis of this type of data gains power if it moves to approaches able to compare the ‘trends’ of the different metabolites. In particular, we show how generalized additive models can be used to model the time-dependent profile of anthocyanin synthesis in grapevine cell suspension cultures (*Vitis vinifera* cv. Gamay), following treatment with 100 μm methyl jasmonate. The sampling was performed daily for 20 days of culturing following elicitation at day 5. All samples were analyzed by UPLC-MS/MS for the identification and quantification of fifteen anthocyanin compounds. The models confirmed the separation in the anthocyanin biosynthetic pathway between delphinidin-based and cyanidin-based compounds, showing that methyl jasmonate modulates the anthocyanin concentration profiles. Our results clearly indicate that the combination of high-throughput metabolomics and state of the art statistical modeling is a powerful approach to study plant metabolism. This approach is expected to gain popularity due to the growing availability of low-cost high-throughput ‘omic’ assays.

## Introduction

Plant cell cultures are model systems to dissect plant biosynthetic pathways and to investigate which factors influence metabolism. Unlike whole organisms, they can be grown under controlled and reproducible conditions and this makes them ideal to collect robust data sets and to develop new methods of data analysis. The treatment of plant cell cultures with biotic or abiotic elicitors often induces the production of secondary metabolites, which in natural conditions are synthesized in response to pathogen attack or environmental stimuli.^1^ A variety of molecules can act as elicitor,^2^ among them, jasmonic acid (JA) and methyl jasmonate (MeJA) own a place of merit. JA and MeJA are fatty acid derived plant hormones that occur ubiquitously in the plant kingdom and act as regulators of defense responses and other plant processes. They trigger a signal transduction chain, which in turn activates multiple secondary biosynthetic pathways.^3^ When exogenously applied to plant cell cultures in the concentration range from 10 to 200, MeJA μm leads to an increased production of secondary metabolites including alkaloids, volatile terpenes and anthocyanins.^4,5,6^ Anthocyanins are a class of phenylpropanoids responsible of the red, blue or purple color of grapes and thus of wine. They are widely studied for their antioxidant properties, free radical scavenging potential and suppression of proliferation of human cancer cells, as well.^7^ All grape anthocyanins derives from five main anthocyanidin skeletons, cyanidin (Cy), delphinidin (Dp), peonidin (Pn), petunidin (Pt) and malvidin (Mv) and might undergo further chemical modifications such as glycosylation or acylation.^8^ Several studies aiming to optimize the *in vitro* production of anthocyanins in *V. vinifera* cell cultures, used MeJA as elicitor,^9,10^ but although considerable progress has been made in the study of the anthocyanin biosynthetic pathway in grapevine,^9^ a deep knowledge of the time-dependent production of the different anthocyanins in response to MeJA elicitation is lacking.

The recent advances in analytical technologies make now possible to use high-throughput metabolomics to investigate the cellular metabolic response over time, so we used targeted metabolomics to obtain a detailed metabolic characterization over twenty days of MeJA elicited Gamay cell cultures. To fully capture the whole information within this type of data, we implemented a new approach using generalized additive models (GAMs).^11^ GAMs are regression models which allow for the inclusion of a non-parametric smoothing and will fit a regression spline to the data, allowing for nonlinear relationships. The level of complexity (nonlinearity) of each term of the model is determined by the estimated degrees of freedom (e.d.f.) of the smoother. An e.d.f.=1 speaks of an almost linear relationship, while e.d.f.>1 indicate a nonlinear relationship.^11^ A standard data analysis looking for significant differences at each individual time point for each metabolite would indeed not capture the ‘longitudinal’ structure of the data set suffering of multiplicity issues.^12^ The central idea of the present paper is to propose a method to perform data analysis at the level of trends. Considering that biological interactions could result in complex time-dependent behaviors, these trends should be fitted with flexibility and in this respect GAMs represent an ideal tool because they are by nature data-driven instead of model-driven. Moreover, their additive structure helps the interpretation phase since each predictor term enters the model separately.^13^ Of course, the need of data points is the price to pay for this flexibility and GAMs are not suitable for investigations dealing with only a limited number of time points.

In this paper, we illustrate how GAMs can be applied to the modeling of targeted metabolomic data in order to address the following questions:

- Are there specific trends in the synthesis of anthocyanins over time?
- Is the addition of MeJA responsible for a change in the production of anthocyanins?
- Is MeJA affecting the synthesis of anthocyanin derivatives differently?

## Materials and methods

### Plant cell line and suspension subcultures

The cell suspension line of *V. vinifera* cv. Gamay Fréaux was a gift of Dr Francosis Cormier’s group^14^ it has been maintained at the Fondazione Edmund Mach in San Michele all’Adige, Italy. The cell culture was cultivated on B5 medium (Gamborg B5 Medium B5VIT, Duchefa B.V., The Netherlands) supplemented with 30 g L^−1^ sucrose, 250 g L^−1^ casein hydrolysate (Merck, Darmstadt), 0.1 mg L^−1^ α-naphthaleneacetic acid (NAA) and 0.2 mg L^−1^ kinetin (K) and 0.8% agar. Callus cultures were transferred every 28 days onto fresh solid sterile medium. The most red pigmented cell aggregates were selected for growth in liquid suspension cultures, obtained by transferring cell aggregates into 50 mL of liquid B5 medium in 250 mL Erlenmeyer flasks, continuously agitated on a rotary shaker at 110 r.p.m. at 25±2 °C under continuous light. The initial pH of the medium was adjusted to 5.5 with 0.1 m KOH before autoclaving. Cells were subcultured every 7 days with an inoculum dilution of 1:3.

### MeJA treatment of the cell cultures

For the elicitation experiment, 7-day-old cells were transferred to the 1 L Erlenmeyer flasks containing fresh B5 medium with the dilution of 1:3 and the total volume for each flask was 450 mL. The cultures were grown under the same conditions as described above. MeJA (Sigma Aldrich, Milan, Italy) was dissolved in 100% EtOH and added at 100 μm final concentration to the 5-days old cultures. Control cultures were given the same volume of a blank solution of ethanol to disentangle the effect of MeJA from the one of ethanol; in all flasks ethanol concentration did not exceed 0.05% (v/v). Triplicate biological replicates were prepared for both control and elicited samples. For the time course effect of MeJA, the cells were harvested every day for 20 days culture period from the same flasks.

### Determination of cell growth and total anthocyanin concentration

For determining cell growth and anthocyanin accumulation, 10 mL of cell suspension cultures from each flask were harvested daily, filtered by vacuum filtration through filter paper (Whatman-Sigma Aldrich, Milan, Italy) and weighed to obtain fresh cell weight. All samples were stored at −20 °C. Anthocyanins were extracted from 100 mg fresh cells. To each sample, 750 μl of 79% (v/v) ethanol with 1% (v/v) glacial acetic acid (extraction solvent) was added, and samples were incubated in a heat block at 85 °C for 20 min. After centrifugation at 13 000 r.p.m. for 5 min, the supernatants were collected, and the pellets were re-extracted with 600 μL of extraction solvent twice. Supernatants were combined, and 50 μL of 37% (v/v) hydrochloric acid was added to stabilize the anthocyanins. After 10  min incubation in the dark at room temperature, the sample was diluted 1:1 (v/v) with the extraction solvent. Total anthocyanin content was determined by measuring absorbance at 535 nm using *ε*=98.2 (dilution factor=2).^15^ For compound identification, the extracts were analyzed by UPLC-MS/MS. To minimize possible analytical artifacts, all the samples were extracted and analyzed at the end of the experiment in a single batch.

### UPLC analysis, identification and quantification (MS/MS) of anthocyanin compounds

Analytical separation of anthocyanin compounds was performed in an Acquity Ultraperformance Liquid Chromatographic (UPLC) system (Waters, UK). The system was coupled to a Waters Xevo TQ MS (Milford, MA, USA) equipped with an electrospray (ESI) source. All samples were analyzed on a reverse phase Acquity UPLC BEH C18, 1.7 μm, 2.1×150 mm column (Waters), protected with an Acquity UPLC BEH C18, 1.7 μm, 2.1×5 mm precolumn (Waters) at 40 °C and under mobile phase flow rate of 0.4 mL min^−1^. Water was used as weak eluting solvent (A) and methanol as strong eluting solvent (B); formic acid 5% v/v was used as additive in both eluents. The multistep linear gradient used was as follows: from 95 to 60% A for the first 4 min, from 60 to 45% A from 4 to 9 min, from 45 to 5% A from 9 to 11 min, and an isocratic hold for 3 min to clean the column. The equilibration time was 4 min, and the injection volume was 2 μL. A quality control standard mixture was injected periodically. All the anthocyanin compounds were detected by multiple reaction monitoring (MRM) by screening the MS/MS transitions and using the parameters earlier optimized for grape wine.^16^ For quantification, external calibration curves were prepared by injecting authentic standards of each compound at different concentrations.

### Data analysis and statistical modeling

All statistical analyses were performed inside the R statistical environment,^17^ and ‘ggplot2’ package was used for visualization.^18^

Principal component analysis (PCA) was performed on mean-centered and scaled data.^19,20^ Pearson correlation was used to assess the similarity between the accumulation patterns of different anthocyanins and the results were visualized using the ‘corrplot’ package.^21^ GAMs were fitted by using the ‘mgcv’ package.^22^ GAMs were fitted with a log link with the Gamma family by using the restricted maximum likelihood (REML) algorithm because REML estimates are more nearly unbiased^23^ in presence of small data sets. The homogeneity of variance across the variable range (homoscedasticity) is one of the assumption in statistical modeling and in general this assumption simplifies mathematical and computational treatment. Metabolite concentrations (as counts derived measures) are usually not homoscedastic logarithm transformation was used to correct for that.^24^

All models were inspected for the presence of heteroscedasticity in the residuals by using the diagnostic plots provided by mgcv. The diagnostic plots are included in the Supplementary Information.

To highlight the differences in the time courses induced by the treatment, in all cases the time-dependent curves were modeled as a common smoother accounting for the ‘general’ time trend, plus a second smoother fitted only for the MeJA treated cultures. An additional treatment factor was used to account for possible constant shifts.

The structure of such type of GAM is the following:

$$C_{\mathrm{Antho}}\left(t\right)=\mathrm{Treatment}+s_{\mathrm{cm}}\left(t\right)+s_{\mathrm{MeJA}}\left(t\right)+\varepsilon$$

Where,

- *C*_Antho_(*t*) is concentrations of anthocyanins over time
- *s*_cm_(*t*) is a smoother that accounts for a common trend in the concentration of anthocyanins over time, both in control and treated cell cultures
- *s*_MeJA_(*t*) is a smoother that accounts for the ‘difference’ in trend for the samples which are treated with MeJA.
- ε is the error.

This type of model is optimal to highlight the effect of the treatment on the concentration of a metabolite, because the specific effect of the elicitation will be captured by the *s*_MeJA_(*t*) term. Nonetheless, it is not the most favorable to compare the trends of two metabolites. To do that the same approach was used to model the ratios between the measured concentrations.

## Results

### Anthocyanin profile of the grapevine cell cultures

A total of fifteen anthocyanins were identified in all the samples of *V. vinifera* cv. Gamay cells either elicited or not (an example of the typical chromatographic profile is included in the Supplementary Information, Supplementary Figure S1). All anthocyanins were monoglycosides: unmodified (delphinidin—Dp-glu, malvidin—Mv-glu, petunidin—Pt-glu, cyanidin—Cy-glu and peonidin—Pn-glu), acetylated (Dp-ac-glu, Mv-ac-glu, Pt-ac-glu, Cy-ac-glu, Pn-ac-glu) or coumaroylated (Dp-pc-glu, Mv-pc-glu, Pt-pc-glu, Cy-pc-glu, Pn-pc-glu). Their structures are presented in Supplementary Figure S2. A schematic view of the biosynthetic pathway is included in the Supplementary Figure S4.

In terms of relative concentrations, Pn-glu and its derivatives were dominant in all samples, accounting for 46–62% of the total in the control and 43–68% in the MeJA treated cultures. The second most abundant pigment is Cy-glu and its derivatives, with 35–52% and 35–53% respectively. The other three anthocyanins, Dp-glu, Pt-glu, Mv-glu and their derivatives, each ranged from 0.4 to 7% in all the samples. The *p*-coumaryl derivatives represented around 44–80% while non-acylated anthocyanins were 18–50% of all anthocyanins. The contribution of the different classes of pigments to the overall anthocyanin profile varied over the growing period. Cyanidin-based anthocyanins (cyanidins and peonidins) were dominant at the beginning of the culture (lag phase), while delphinidin-based anthocyanins accumulated rapidly from the beginning of the exponential growth phase in both control and MeJA treated cell cultures (Supplementary Figure S3).

### Time course changes of anthocyanin biosynthesis and MeJA treatment

**Figure 1:**
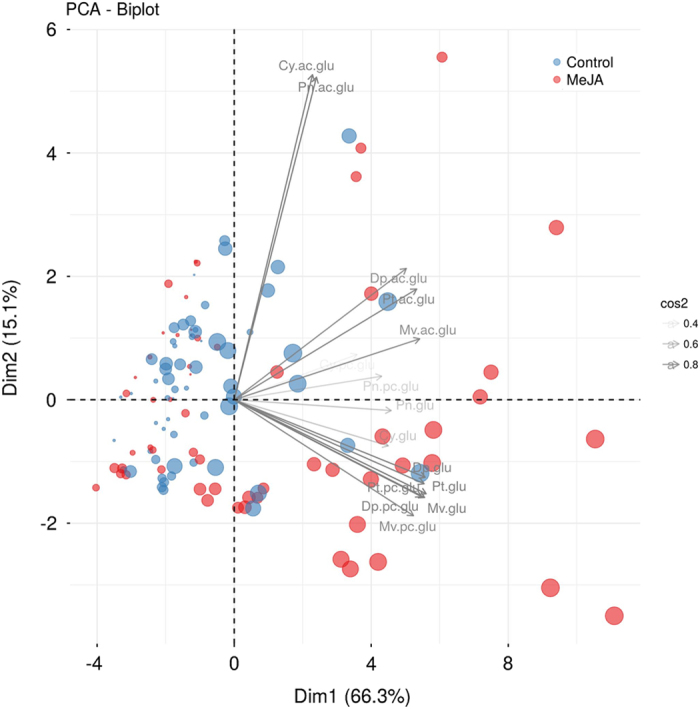
Biplot of the Principal Component Analysis (PCA) on the autoscaled data showing the projection of the data set in the PC1×PC2 plane. The size of each dot is proportional to the sampling time (1–18 days). The gray arrows show the contribution of the metabolites to each principal component. Transparency is used to highlight metabolites strongly contributing to the PC1×PC2 projection.

Principal Component Analysis (PCA) was performed to get a global overview of the effects of MeJA on the anthocyanin profile. Figure 1 shows the projection of the data on the PC1×PC2 plane accounting for 81% of the total variance. PCA does not show a clear separation between treatment and control at the earlier stages. However, the treated samples start to diverge after day 8, indicating that the effect of elicitation on the anthocyanin biosynthesis could be appreciated only after few days from the treatment. PC1 is the direction characterizing time evolution, which then accounts for the bigger part of the total variability.

**Figure 2:**
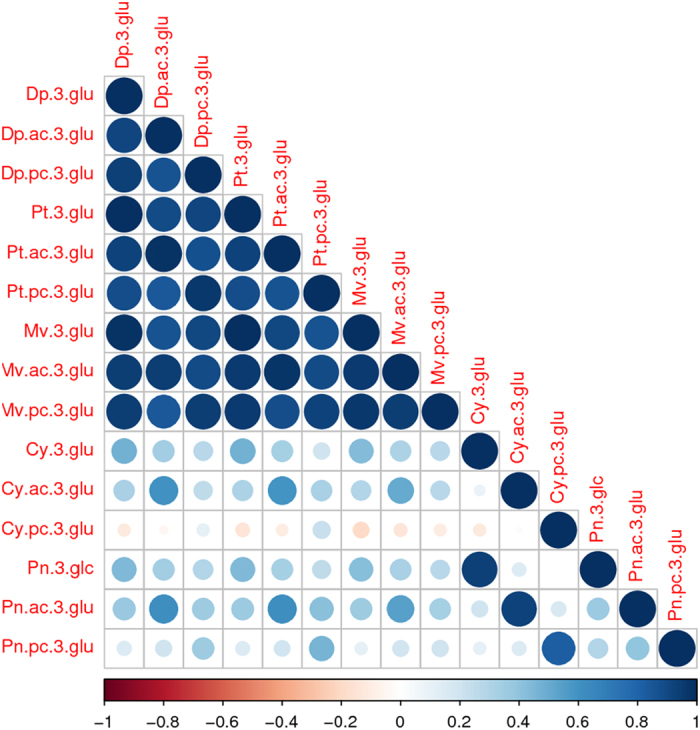
Correlation matrix of all anthocyanins in the control grape cell cultures. The shape and the color of each dot in the triangular matrix show the strength of Pearson correlation (positive or negative) between pairs of anthocyanins. Positive strong correlations stand out in dark blue. Lighter colors indicate weaker relations between the metabolites.

Pairwise correlation analysis was performed to compare the trends of the fifteen anthocyanins and to identify the ones to be included into statistical modeling. Pearson correlation coefficients for anthocyanins in control cell cultures are shown in Figure 2. Dp-glu shows a strong and highly significant positive correlation with Mv-glu, Pt-glu and their acetyl and coumaryl derivatives, in agreement with the fact that Dp is the precursor of Pt and Mv.^25^ In both cases, monoglucosides were positively correlated with their acetyl and coumaryl glucosides within the pathway too. Cyanidins and peonidins seem to be independent from delphinidins. Cy, as a precursor of Pn, was strongly correlated with Pn-glu and the same is true for their acetyl and *p*-coumaryl derivatives. Remarkably, no strong correlations were instead found between monoglucosides and their respective acetyl and coumaryl glucosides for both cyanidins and peonidins. Correlation analysis shows that anthocyanin production followed two distinct pathways: one typical of delphinidins and the other typical of cyanidins. On the basis of the correlation analysis Dp-glu, Cy-glu and their derivatives were taken as ‘representative’ for the subsequent statistical modeling.

### Total anthocyanin modeling

**Figure 3:**
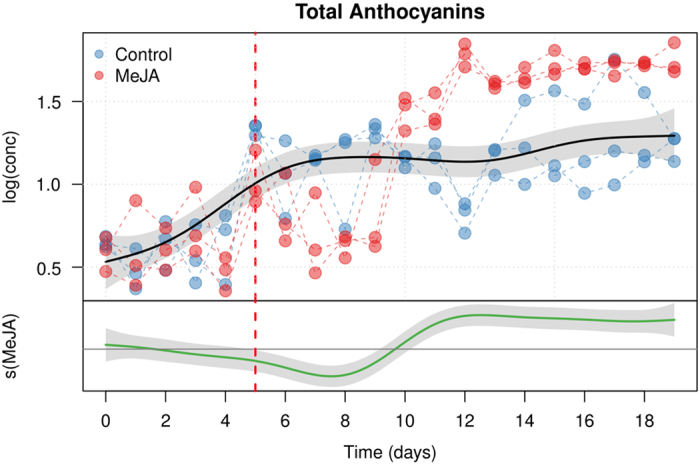
Modeling of the time-dependent concentration of anthocyanins in *V. vinifera* (L.) cv. Gamay cell suspensions elicited with MeJA. The upper plot shows the fitted smooth of the common trend (*s*_*cm*_*(t)*), while the lower graph (MeJA) shows the additional effect of the MeJA term (*s*_*MeJA*_*(t)*). The shaded area of the trends are the 95% confidence intervals of the fitted smoothers. The day of MeJA elicitation is indicated by a red dashed line.

The model showing the effect of MeJA treatment on the total anthocyanin synthesis is depicted in Figure 3. The common trend line (e.d.f.=3.293, *P*<0.0001) indicates a continuous increase of total anthocyanin content in the cells during the first 7 days of growth, followed by a stable anthocyanin concentration. The bottom model component shows instead the ‘additional’ effect of MeJA. The two cultures start to diverge after day 9 and the difference in concentration stabilizes around day 13. A slight decrease in the in anthocyanin concentration seems to be present between day 5 and day 9.

### Glycosylated anthocyanin modeling

**Figure 4:**
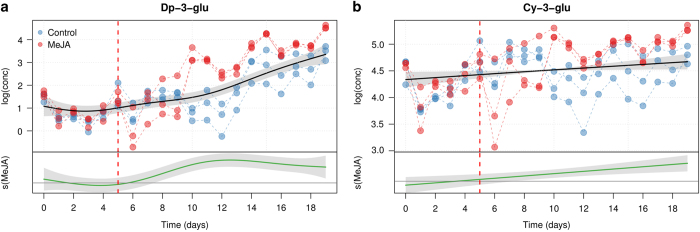
GAM modeling of the concentration of Dp-3-glu (**a**) and Cy-3-glu (**b**). For each panel, the upper plot shows the fitted smooth of the common trend (*s*_*cm*_*(t)*), while the lower graph (MeJA) shows the additional effect of the MeJA term (*s*_*MeJA*_*(t)*). The shaded area of the trends are the 95% confidence intervals of the fitted smoothers. The day of MeJA elicitation is indicated by a red dashed line.

The effect of time and treatment on Dp-glu and Cy-glu is presented in Figure 4. From the plots it is clear that the relative contribution of the two metabolites evolves over time. The common smoother for both metabolites show an increase over time. The trend for Dp-glc is strongly significant (e.d.f.=3.701, *P*<0.0001), while the one for Cy-glu is less clear (e.d.f.=1.08, *P*=0.0284). On this respect it is worth mentioning that *P*-values calculated for GAMs are only approximate, so a *P*-value of 0.02 does not fully support the significance of the observed trend.

The two differential smoothers clearly indicates that MeJA influenced significantly the biosynthesis of both glucosides. In particular, the maximum concentration of Dp-glu in the MeJA cell cultures reached 94.74 ±7.11 μg/mg (mean±s.d.), and was three times higher than in the control culture at day 20. The effect on the other branch of the pathway was smaller, with a maximum of 198.81±11.46 μg mg^−1^ (mean±s.d.) of Cy-glu in the MeJA treated cells, which is 1.63 times higher than in the control culture at day 19.

### Acylated anthocyanins modeling

**Figure 5:**
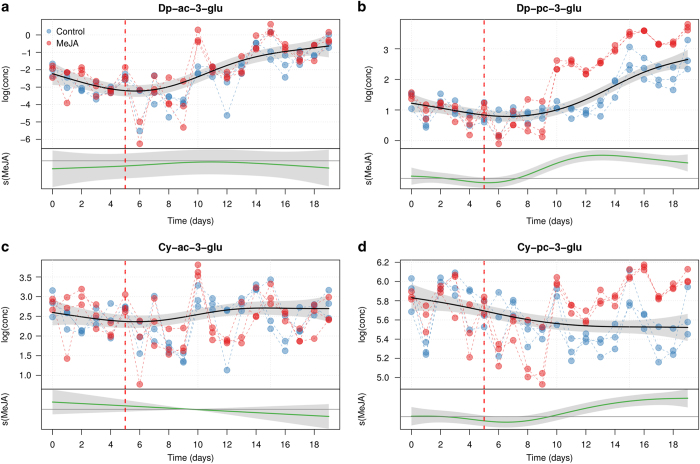
GAM modeling of the concentration of the acylated derivatives of Dp-3-glu and Cy-3-glu (Dp-ac-3-glu (**a**), Dp-pc-3-glu (**b**), Cy-ac-3-glu (**c**), Cy-pc-3-glu (**d**)) For each panel, the upper plot shows the fitted smooth of the common trend (*s*_cm_*(t)*), while the lower graph (MeJA) shows the additional effect of the MeJA term (*s*_MeJA_*(t)*). The shaded area of the trends are the 95% confidence intervals of the fitted smoothers. The day of MeJA elicitation is indicated by a red dashed line.

**Figure 6:**
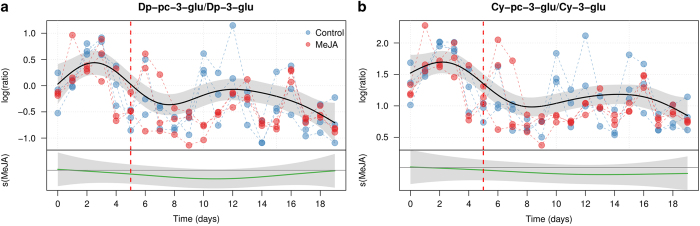
GAM modeling of the ratios between the measured concentration of Dp-pc-3-glu and Dp-3-glu (**a**), and Cy-pc-3-glu and Cy-3-glu (**b**). For each panel, the upper plot shows the fitted smooth of the common trend (*s*_cm_*(t)*), while the lower graph (MeJA) shows the additional effect of the MeJA term (*s*_MeJA_*(t)*). The shaded area of the trends are the 95% confidence intervals of the fitted smoothers. The day of MeJA elicitation is indicated by a red dashed line.

The effects of growth and elicitation on the production of acetylated and coumarylated anthocyanins are displayed in Figures 5 and 6. In terms of absolute concentrations, acetylated anthocyanins were present in lower amounts in both control and treated cell cultures as compared to coumarylated ones. The common trend of Dp-ac-glu biosynthesis in the cells shows a significant nonlinear increase over time (e.d.f.=3.48, *P*<0.001). However, the differential smoother is not significant (e.d.f.=3.28, *P*=0.55) with large 95% confidence interval bands, indicating that MeJA elicitation had no effect on the concentration of Dp-ac-glu. This result was confirmed for Cy-ac-glu: the concentration of this metabolite is indeed neither changing with time (common, e.d.f.=2.973, *P*=0.161), nor affected by MeJA treatment (differential, e.d.f.=2.000, *P*=0.505). Also the concentration of Dp-pc-glu shows an increase over time, but here the effect of MeJA is much stronger. The differential smoother for Dp-pc-glu, indeed, is significant (e.d.f.=8.42, *P*<0.0001), as in the case of Cy-pc-glu.

The data presented in Figures 4 and 5 indicate that the treatment with MeJA affects the two glucosides (Dp-3-glu and Cy-3-glu) and their p-coumaryl derivatives. It is then interesting to assess if the effect of MaJA is the same on the two classes of metabolites. To do that GAMs were used to model the ratios between the concentrations two glucosides and their p-coumaryl derivatives in the two branches of the pathway. The outcomes of the modeling ratios are shown in Figure 6. In both cyanidin and delphinidin branches the results are similar. The common trends (Dp-pc-glu/Dp-glu, e.d.f.=7.065, *P*<0.001 and Cy-pc-glu/Cy-glu e.d.f.=5.153, *P*<0.001) are significant and show a similar decrease over time, speaking of an effect of culturing time on this metabolic conversion. On the contrary, the differential smoothers are not significant (e.d.f.=2.854, *P*=0.477) indicating that MeJA affected the concentration of both metabolites in a similar way.

### Technical approach

**Figure 7:**
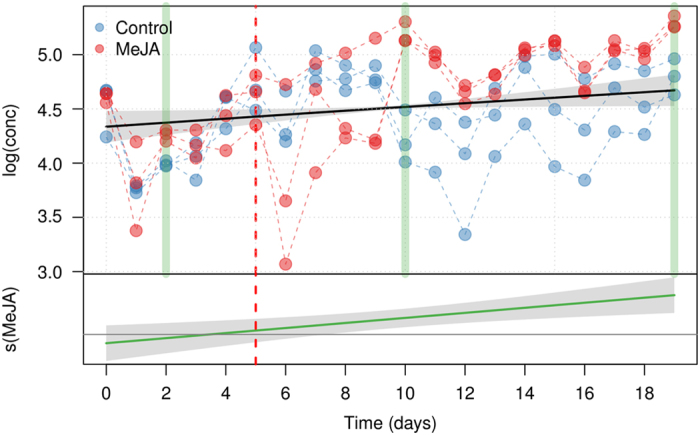
Comparison between GAM analysis and *t*-test performed at each separate time point on the Cy-glu concentration. The vertical green bars highlight the time points where the concentration in treated and untreated cells was significantly different (BH—FDR correction at the 0.01 level). The upper plot shows the fitted smooth of the common trend (*s*_*cm*_(t)), while the lower graph (MeJA) shows the additional effect of the MeJA term (*s*_*MeJA*_(t)). The shaded area of the trends are the 95% confidence intervals of the fitted smoothers. The day of MeJA elicitation is indicated by a red dashed line.

The results here presented clearly indicate that the use of appropriate time course metabolomics experiments is crucial to interpret metabolic profiles since in plants metabolic levels change dynamically.^26^ However, they also demonstrate that to analyze these type of experiments it is necessary to develop *ad hoc* data analysis strategies. To further highlight this fact, Figure 7 compares the outcomes of a statistical analysis performed at each separate time point with the ‘global’ one allowed by GAMs. In the first case, the effect of MeJA on the Cy-glu synthesis would be considered significant only at three (non consecutive) sampling points and this would suggest a small and somehow erratic effect of MeJA on this metabolite. The GAMs are telling a different story suggesting a smooth and consistent effect, more in line with the expected biological response and the real nature of the experimental data.

GAMs, being a fitting model, are neutral with respect to the application and thus their use is not limited to the analysis of metabolic data. They are effectively applied each time it is required to analyze the dependence between two variables and there is no *a priori* knowledge about the type of dependence. In this specific study we investigated the dependence of anthocyanin concentration on time or elicitation with MeJa and we did not know whether this was modeled by a specific analytical function. As mentioned in the introduction GAMs can be fitted when a high number of data points are available, usually ten or more. It is important to remark that the knowledge about the structure of the pathways is not a requirement in order to apply the method, but clearly this knowledge will help in interpreting the results of the analysis. A change of the concentration of a metabolite is indeed always a balance between its rate of synthesis and rate of consumption and this balance can be not straightforward in the case of connected and/or circular pathway structures.

## Discussion

The general finding of our investigation is that the concentration patterns of the anthocyanins are changing in time to a different extent depending on their position in the biosynthetic pathway and that the elicitation by MeJA selectively changes these trends.

At the global scale, the composition of anthocyanin compounds found in our work is in agreement with other studies.^27,28^ Anthocyanins biosynthesis in *V. vinifera* species is indeed genetically controlled and involves two main pathway branches, namely the delphinidin-based (3’,5’ OH) and the cyanidin-based (3’OH).^25,29^ In terms of time trends, our study demonstrates that the total anthocyanin content increased over time mainly due to an increased concentration of Dp-glu and its derivatives (Dp-ac-glu and Dp-pc-glu) and that the elicitation by MeJA further enhanced this behavior. The increase in anthocyanin content was expected as it has been reported in several studies with grapes and grape cell cultures.^10, 30,31,32,33^

Looking at the individual anthocyanins, Dp-glu and its derivatives (Dp-ac-glu and Dp-pc-glu) increased over time whereas Cy-glu and its derivatives remained constant. The non-significant changes of Dp-pc-glu/Dp-glu and Cy-pc-glu/Cy-glu might be the consequence of coumarylated glucosides being produced at the same rate of non-acylated glucosides. However, a different rate of consumption of the two molecules can not be ruled out and it would lead to the same result: coumaroyl-glucoside derivatives may be more reactive and preferential substrate for the formation of derived pigments. A higher reactivity has been indeed previously reported in a fermentation study, where the rate of synthesis of *p*-coumaroyl-vitisins resulted higher than that of non-acylated vitisins.^34^

It is interesting to focus on the effect of MeJA on the ‘balance’ between the four acylated anthocyanins and their relative glycosylated precursors. After treatment, indeed, coumarylated derivatives ‘were following’ the trend of their glycosylated precursor, while their acetylated counterparts were not. Remarkably, this was happening both in the Dp and in the Cy branch of the pathway. These results suggest that the treatment with MeJA could selectively modulate the activity of specific acyltransferases that can use both glycosylated forms as precursor. Our findings are interesting because acylation is one of the most common modifications of anthocyanins,^8^ but the mechanism controlling the balance between the pool of non-acylated and acylated forms is still unknown. The presence and the specific activities of the enzymes involved in these reactions are strongly related to the genetic background of the different grape varieties^35^ and indeed anthocyanin patterns are grape variety specific. Our results somehow complicate this picture since we are also showing that these profiles can be modulated by metabolic factors.

The data presented in the paper clearly show the advantages of GAMs as a statistical tool for the analysis of time-resolved metabolomics data and the conceptual gain in moving to the non-parametric analysis of trends. In presence of more complex biological pathways, clear-cut mechanistic understanding will also require the implementation of more insightful experimental strategies like isotopic labeling and time-resolved gene expression analysis to fully characterize metabolic fluxes.^36,37,38^ The general statistical framework will be nonetheless the same. We believe that this type of data analysis strategy has strong potential for the interpretation of the data coming from a wide class of omic time-resolved experiments, which will gain popularity due to the growing availability of low-cost high-throughput ‘omic’ assays.
