# Supplementary material for: Applying generalized additive models to unravel dynamic changes in anthocyanin biosynthesis in methyl jasmonate elicited grapevine (Vitis vinifera cv. Gamay) cell cultures
Source: Hortic Res. 2017 Jul 26;4:17038–. doi: 10.1038/hortres.2017.38 (PMC5527128; doi:10.1038/hortres.2017.38)

Applying Generalized Additive Models to Unravel Dynamical Changes in Anthocyanin Biosynthesis in Methyl Jasmonate Elicited Grapevine (*Vitis vinifera* cv. Gamay) Cell Cultures

Running Title: Modeling secondary metabolism in cell cultures

Nay Min Min Thaw Saw, Claudio Moser, Stefan Martens, Pietro Franceschi*

Research and Innovation Centre, Fondazione Edmund Mach (FEM), Via E. Mach 1,

38010 San Michele all'Adige, Italy.

* Correspondence to

Pietro Franceschi: pietro.franceschi@fmach.it

Tel: +39-0461-615556

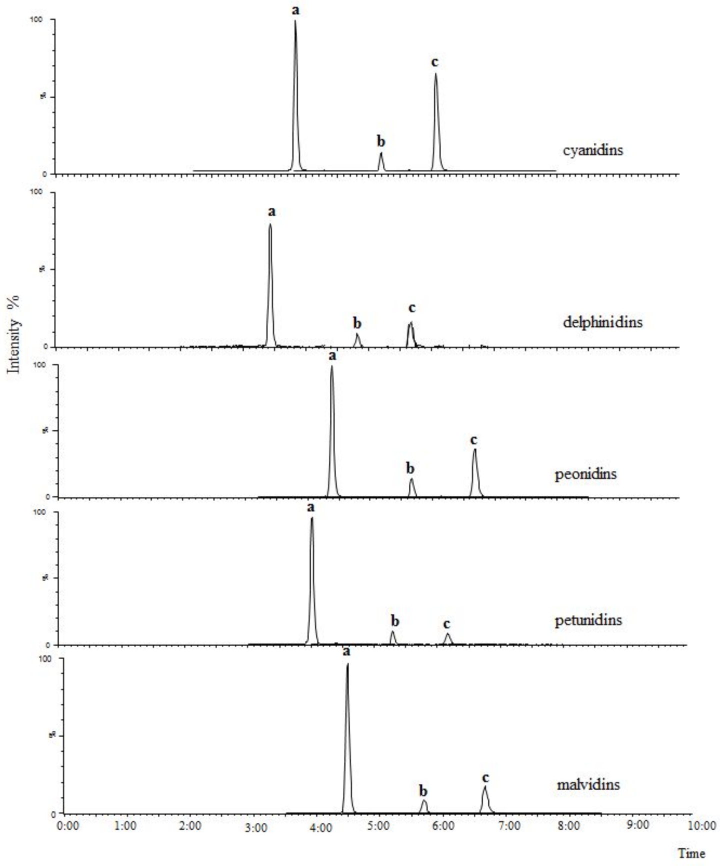

Figure S1: Fig 1. UPLC/QqQ-MS/MS chromatograms of anthocyanins; monoglucosides (a), acetyl glucosides (b) and coumaryl glucosides (c) obtained from grape cells extract.

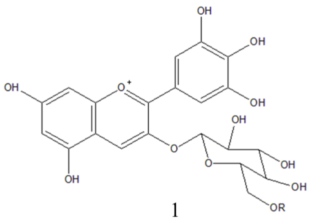

1

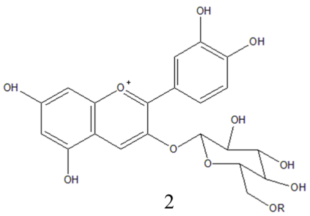

2

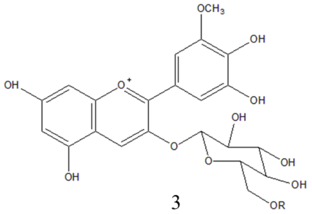

3

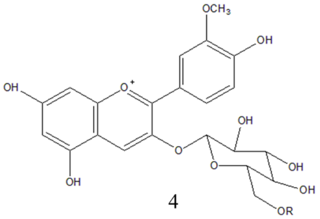

4

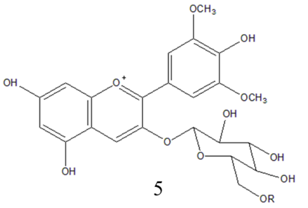

5

Figure S2: Fig 2. Principal anthocyanins in V. vinifera (L.) cv. Gamay cell suspension cultures. R: H, acetyl, p-coumaroyl; (1) delphinidin-3-O-glucoside, (2) cyanidin-3-O-glucoside, (3) petunidin-3-O-glucoside, (4) peonidin-3-O-glucoside, (5) malvidin-3-O-glucoside

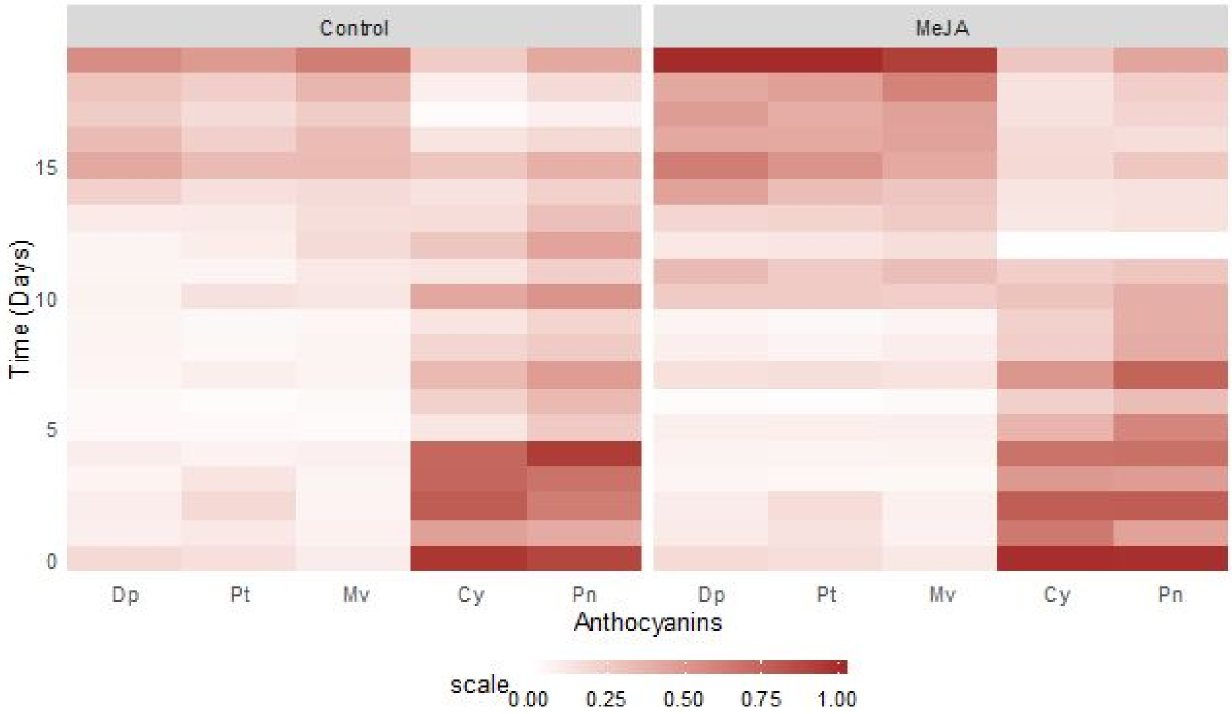

Figure S3. Anthocyanin composition in percentage of total anthocyanins of Vitis vinifera (L.) cv Gamay cell suspensions applied with MeJA

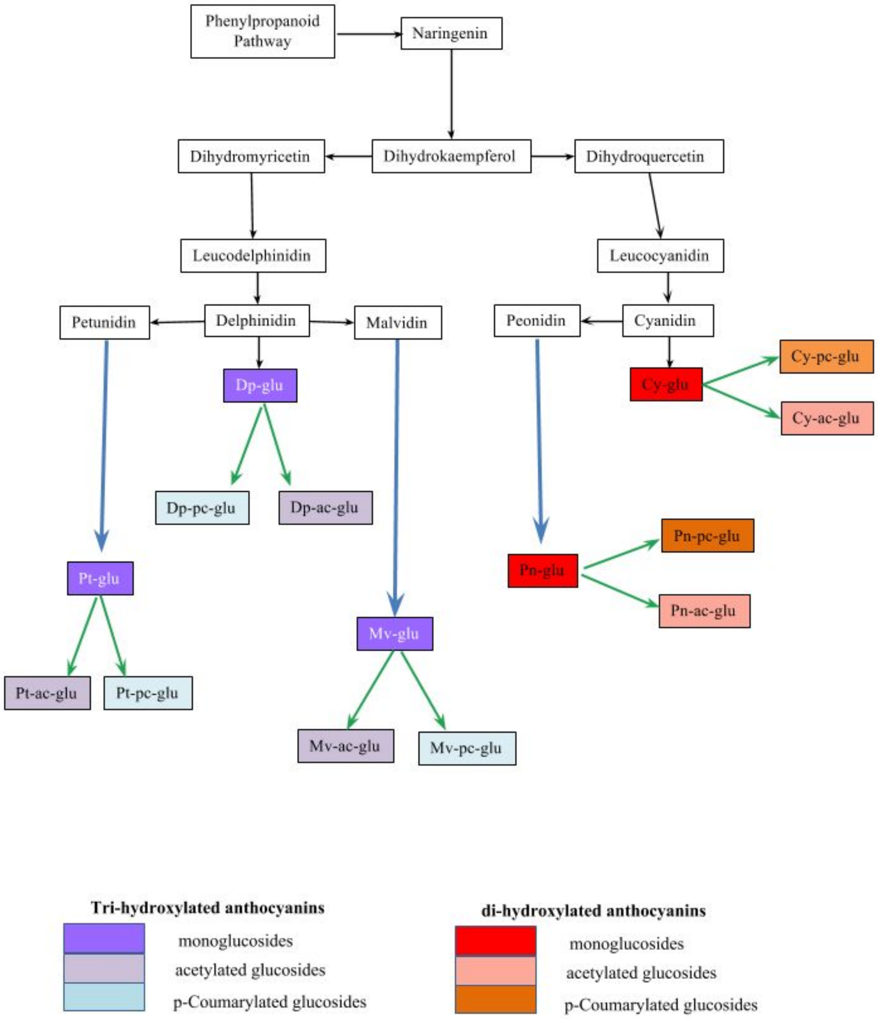

Figure S4 Scheme of the biosynthesis pathway of anthocyanins in grape cell cultures: Dp-glu: Delphinidin-3-O-glucoside; Dp-ac-glu: Delphinidin-acetyl-3-O-glucoside; Dp-pc-glu: Delphinidin-3-O-(6-p-coumaroyl)glucoside.Cy-glu:Cyanidin-3-O-glucoside; Cy-ac-glu:Cyanidin-acetyl-3-O-glucoside;Cy-pc-glu:Cyanidin 3-O-(6-p-coumaroyl)glucoside; Pt-glu:Petunidin-3-O-glucoside; Pt-ac-glu:Petunidin-acetyl-3-O-glucoside; Pt-pc-glu: Petunidin 3-O-(6-p-coumaroyl)glucoside; Mv-glu: Malvidin-3-O-glucoside; Mv-ac-glu: Malvidin-acetyl-3-O-glucoside; Mv-pc-glu:Malvidin 3-O-(6-p-coumaroyl)glucoside; Pn-glu: Peonidin-3-O-glucoside; Pn-ac-glu: Peonidin-acetyl-3-O-glucoside; Pn-pc-glu: Peonidin 3-O-(6-p-coumaroyl)glucoside.

Diagnosis of GAM models

Total anthocyanin concentration

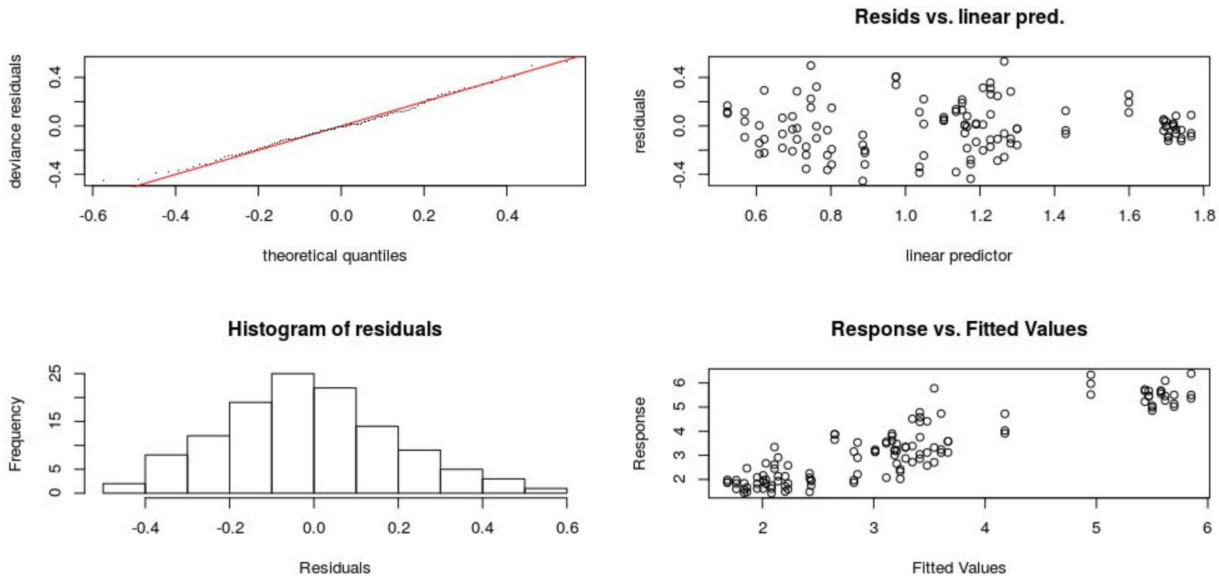

Delphinidin-3-O-glucoside

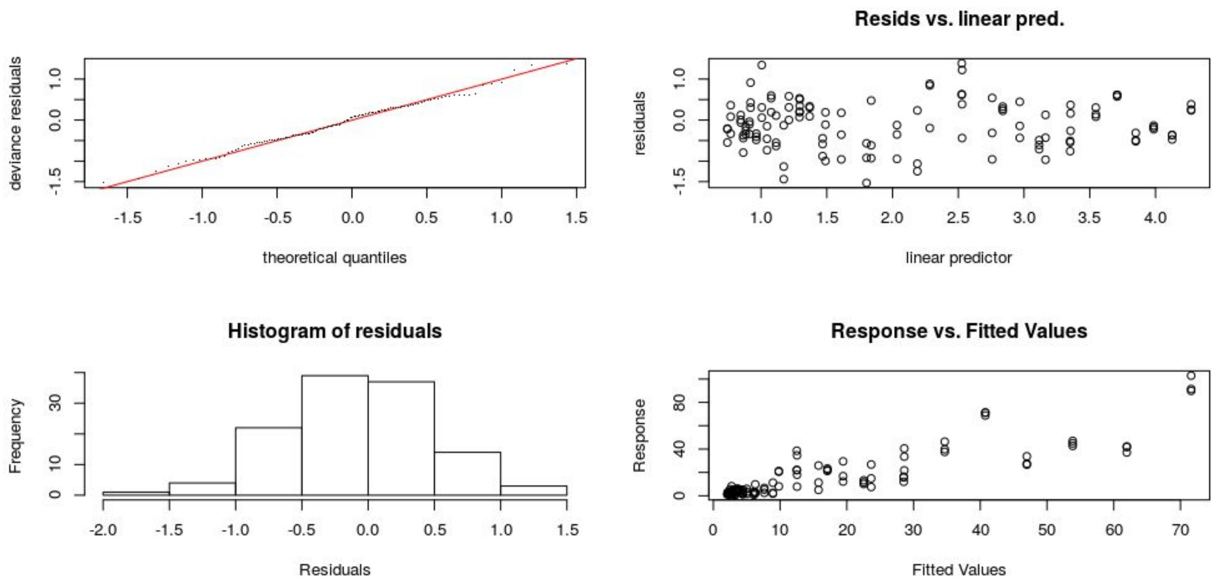

Cyanidin-3-O-glucoside

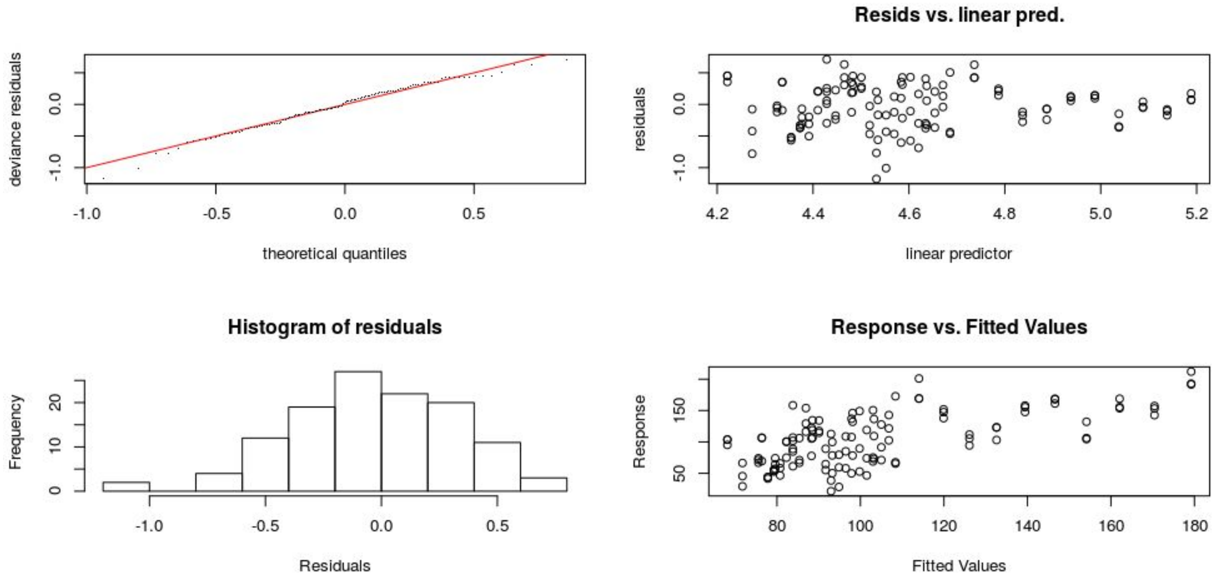

Delphinidin-Ac-3-O-glucoside

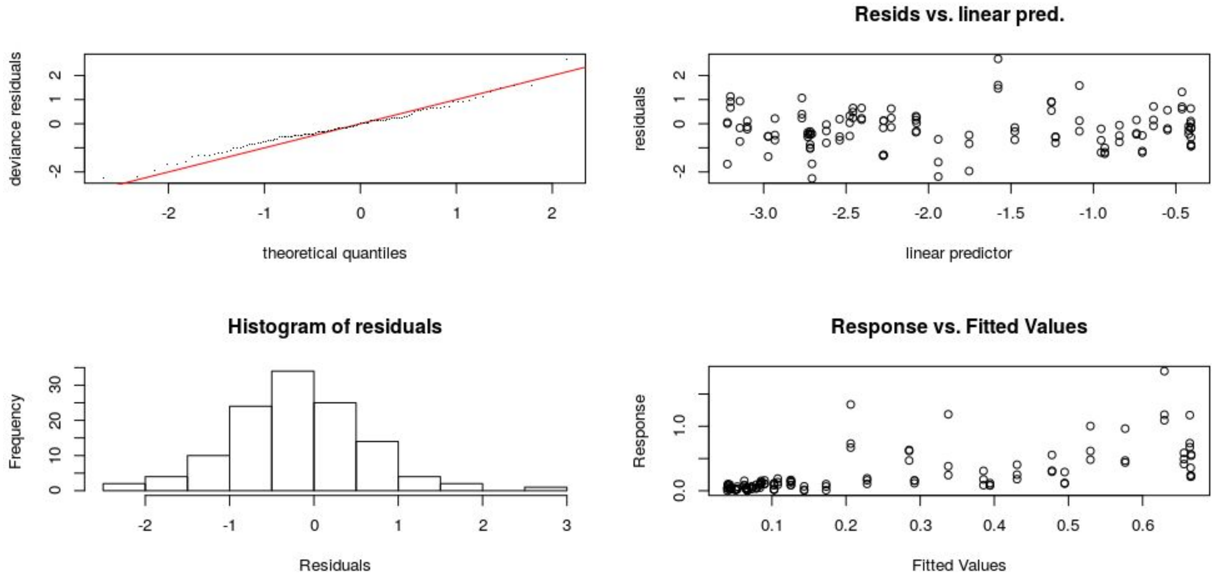

Delphinidin-pc-3-O-glucoside

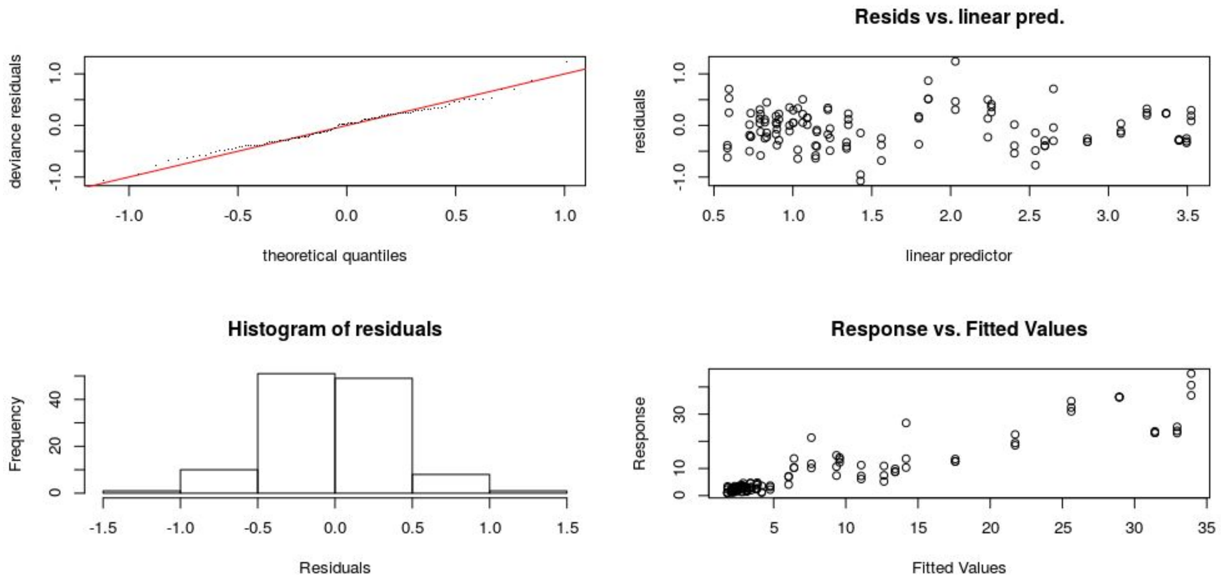

Cyanidin-Ac-3-O-glucoside

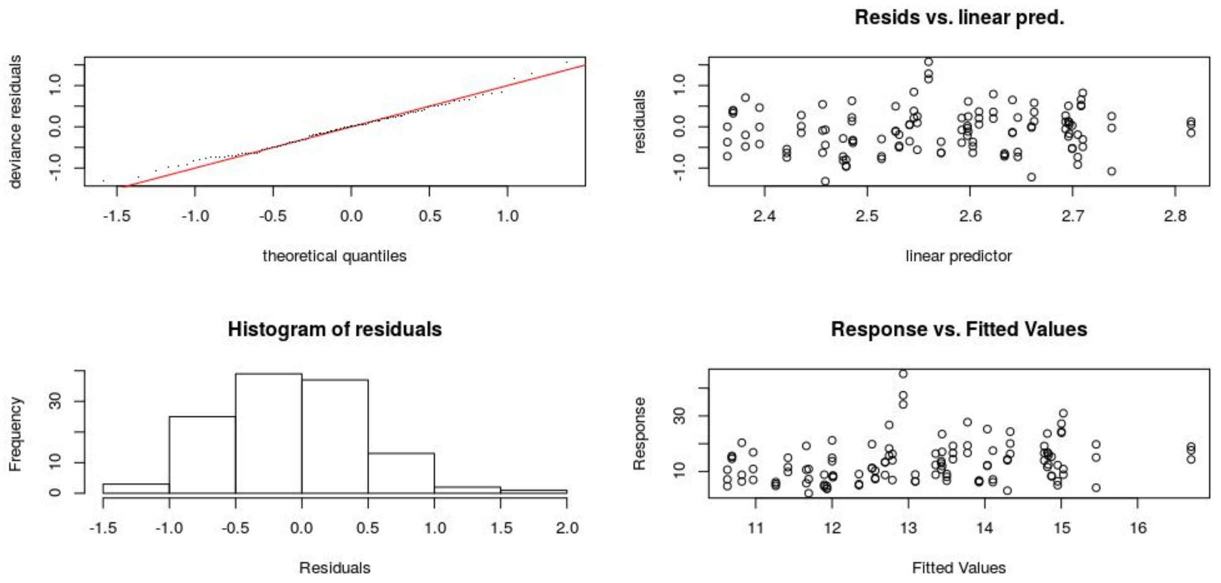

Cyanidin-pc-3-O-glucoside

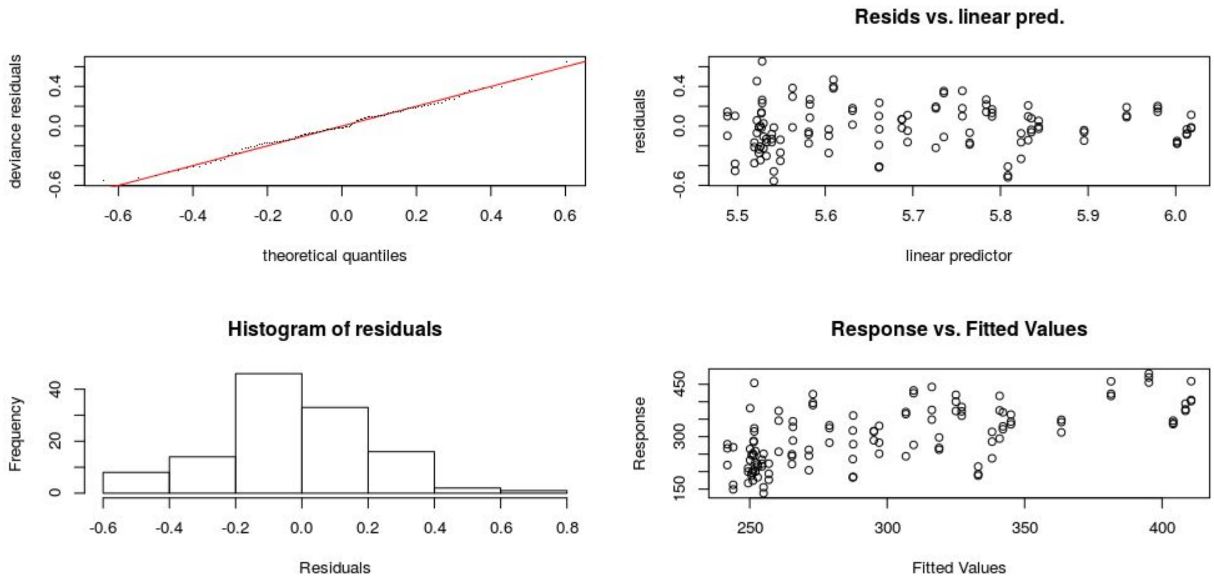

Cyanidin-pc-3-O-glucoside/Cyanidin-3-O-glucoside

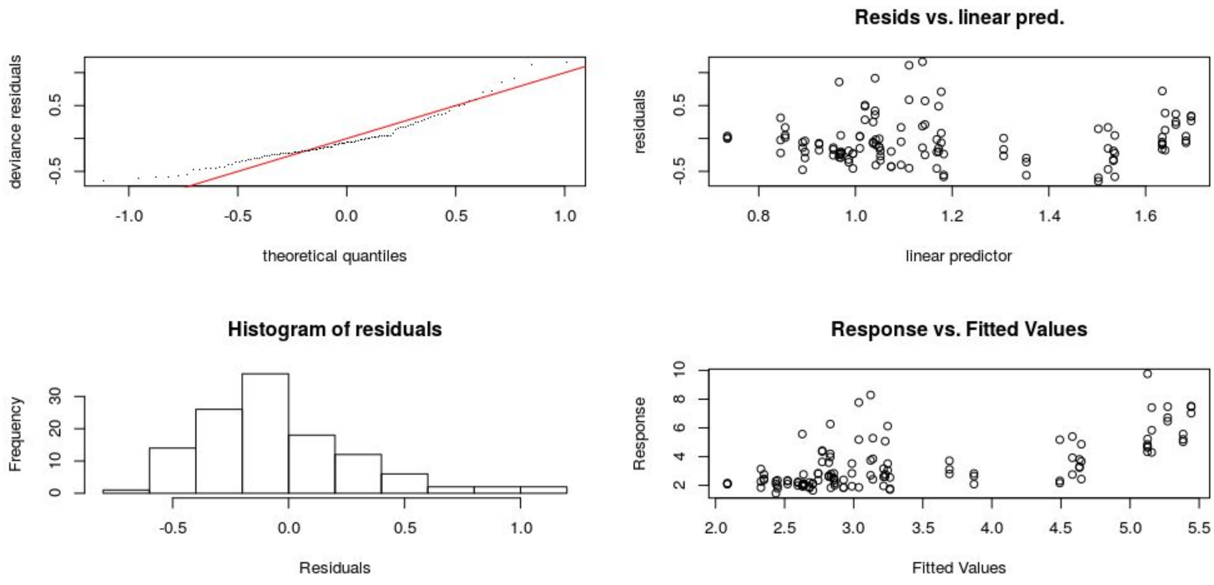

Delphinidin-pc-3-O-glucoside/Delphinidin-3-O-glucoside

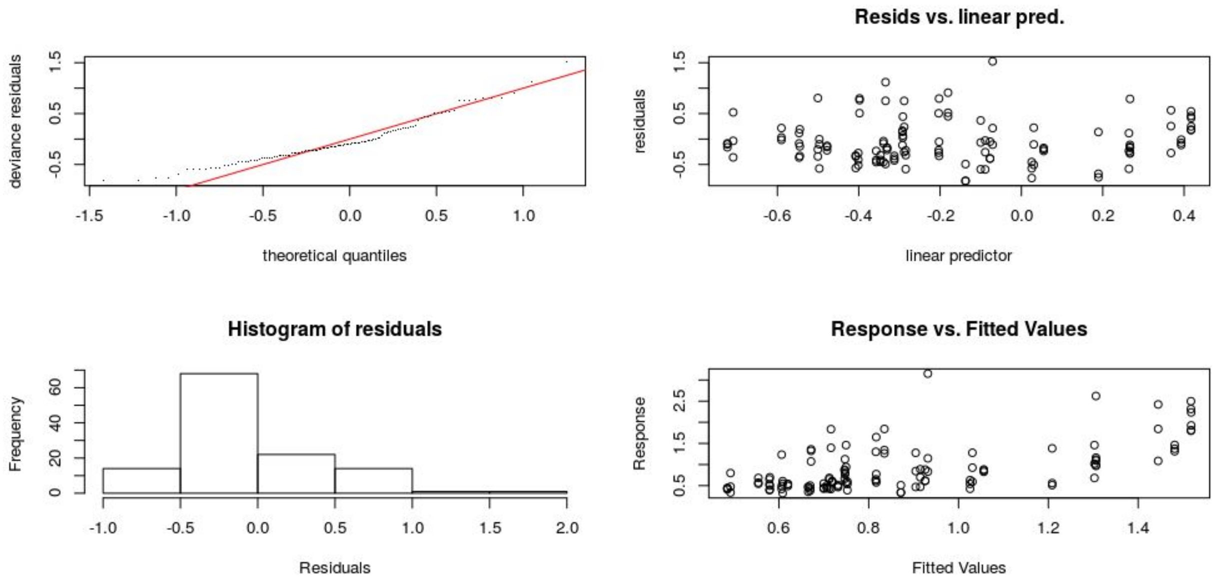

Supplement: Supplementary Information [file hortres201738-s1.pdf]
